# Factors associated with uptake of human papilloma virus vaccine among school girls aged 9–14 years in Lira City northern Uganda: a cross-sectional study

**DOI:** 10.1186/s12905-023-02511-z

**Published:** 2023-07-08

**Authors:** Renniter Mirembe Nakayita, Deo Benyumiza, Catherine Nekesa, Ivan Misuk, Julius Kyeswa, Aisha Nalubuuka, Tom Murungi, Samson Udho, Edward Kumakech

**Affiliations:** 1 Department of Midwifery, Faculty of Nursing and Midwifery, Lira University, P.O. BOX 1035, Lira City, Uganda; 2 Department of Nursing, Faculty of Nursing and Midwifery, Lira University, P.O. Box 1035, Lira City, Uganda

**Keywords:** Human Papilloma Virus Vaccine, Uptake, Adolescent School Girls, Lira City

## Abstract

**Background:**

Cervical cancer is the most common Human Papilloma Virus (HPV)-related disease among women. Since 2008, HPV vaccination has been routinely recommended for pre-adolescent and adolescent girls in Uganda as the primary preventive measure for cervical cancer. However, in Uganda, most especially in Lira district, there is limited literature on HPV vaccination uptake and associated factors among girls aged 9-14years. This study assessed the uptake of HPV vaccine and associated factors among in-school girls aged 9–14 years in Lira City, northern Uganda.

**Methods:**

A cross-sectional study was conducted among 245 primary school girls aged 9–14 years in Lira City, northern Uganda. Multistage sampling technique was used to sample eligible participants and data was collected using interviewer administered questionnaire. Data was analysed using SPSS version 23.0. Descriptive statistics and multivariate logistic regression at 95% level of significance were used to identify the level of HPV vaccine uptake and predictors respectively.

**Results:**

HPV vaccination uptake was at 19.6% (95% CI,14.8–25.1) among the school girls aged 9–14 years in Lira City, northern Uganda. The mean age of the girls was 12.11 (± 1.651) years. Predictors that were independently associated with HPV vaccine uptake included; recommendation from health worker [aOR 9.09, 95% CI (3.19–25.88), *P* ≤ 0.001], taught about cervical cancer at school [aOR,12.56, 95% CI (4.60–34.28), *P* ≤ 0.001], and exposure to outreach clinics [aOR, 4.41, 95% CI (1.37–14.19), *P* = 0.013].

**Conclusion:**

The study found that one in five of the school girls in Lira City, northern Uganda. received HPV vaccine. Girls who were taught about cervical cancer at school, exposure to outreach clinics and received health worker recommendation had more odds of receiving HPV vaccine than their counter parts. The Ministry of Health should strengthen school based cervical cancer education, awareness raising about HPV vaccination and health worker recommendations to improve HPV vaccine uptake among school girls in Uganda.

## Study background

Genital human papillomavirus (HPV) infection is the most common sexually transmitted infection worldwide, estimated at 75% and 80% among men and women of all ages respectively [1]. HPV is considered to be one of the most important risk factors in the development of cervical cancer with persistent infection of high-risk HPV oncogenic types 16 and 18 [2]. Globally, cervical cancer is the fourth most common cancer among women, with an estimated 604,000 new cases and 342,000 related deaths in 2020 [3]. Low- and Middle-income countries disproportionately carry a high burden with 90% of the new cases and related deaths due to cervical cancer [3].

Global HPV immunization coverage as of 2018 was estimated at 12.2% [4], despite improvements in HPV immunization programs in the past 15 years. In sub-Saharan Africa HPV vaccine completion is estimated at 20% compared to developed countries such as Australia and New Zealand with 77% completion of HPV vaccine dosage [5, 6]. To achieve the 90-70-90 World Health Organization (WHO) targets by 2030 [7], the WHO recommended that countries on the path of cervical cancer elimination should have 90% of the girls vaccinated before 15 years, 70% of women screened by age of 35 years and again by 45 years of age, and 90% of women identified with cancer receive treatment [7].

In Uganda, cervical cancer is the number one cause of death among women with cancer-related deaths estimated at 40.5 per 100,000 [8]. HPV vaccination has been routinely recommended for pre-adolescents and adolescent girls in Uganda [9]. The introduction of HPV vaccines represents a step forward in the primary prevention of cervical cancer and other HPV-related malignancies. Currently, there are two types of HPV vaccine: bivalent, which mainly protects against HPV genotypes 16 and 18, and Quadrivalent, which protects against genotypes 6, 11, 16, and 18 [10, 11]. The vaccines have been shown to provide cross-protection against other oncogenic HPV genotypes as well [12, 13]. The Ministry of Health Uganda (MoH) has continued to address challenges associated with HPV vaccination delivery strategy since 2015, through multiple vaccination centers such as schools, health facilities, and outreach posts currently providing routine immunization services [14]. Studies show that school attendance is positively associated with HPV vaccination status due to the less effort required to reach out to girls in the implementation of the HPV vaccine delivery strategy [15, 16],

Despite the concerted efforts, previous secondary data analysis study showed that the uptake of HPV vaccine among girls aged 10–14 years was as low as 22% across Uganda [16]. In addition, a previous community-based study among adolescent girls in northern Uganda also indicated low uptake of HPV vaccine [17]. However, these previous studies did not examine school-based health service context and its association with the uptake of HPV vaccine among the school girls in Uganda. Therefore, this study investigated the uptake of HPV vaccine and associated factors among school girls aged 9–14 years in Lira City, northern Uganda to assist in re-designing appropriate strategies meant to enhance the HPV vaccination uptake in Lira City and Uganda as a whole.

## Materials and methods

### Study design and setting

This was a cross-sectional study employing quantitative methods of data collection and analysis. This study was conducted among six schools in Lira City, northern Uganda. Lira City is the main, administrative, and commercial centre of the Lira District. It is located approximately 100 km (62 miles), southeast of Gulu City the largest city in northern Uganda, and approximately 337 km (209 miles), by road, north of Kampala the capital city of Uganda.

### Study population

This study was conducted among 245 girls aged 9–14 years in selected primary schools located in Lira City, northern Uganda. All girls aged 9-14years who were available at the time of data collection studying in selected schools and had assented were included in the study. Written informed consent was obtained from the parents/guardians of the participants/adolescent school girls. Girls who were ill, those whose parents had not consented, and girls who did not assent to participate in the study were excluded from the sample frame before the participant level sampling procedure. This study focused on girls aged 9–14 years because they are the target age group for the HPV vaccine and are also less likely sexually exposed [15].

### Sample size determination

The sample size was determined using the Leslie Kish formula (1965) n = z^2^pq/d^2^ with the assumptions, the confidence interval was taken at 95%., the level of uptake of HPV vaccine taken (p) was 0.1761 [17], the level of those not vaccinated given by (q) was 0.8239, The z-score (z) was 1.96, a margin of error (d) was 0.05. Therefore, the calculated sample size plus 10% of the non-response was 245 participants.

### Data collection method and tools

We used researcher-administered questionnaires to collect data. The questionnaire was developed by the research team, piloted, reviewed, and adjusted accordingly to fit our study objectives before the actual data collection process. The content validity index (CVI) of the tool was found to be 80% during pretesting which was acceptable. Being a questionnaire not a scale, reliability indices analyses were unnecessary. The questionnaire collected information on the following: demographic characteristics of participants (age, class, tribe, religion, birth order, staying with parents, family type), uptake of the vaccine (having received the HPV vaccine, if yes, how many times, if no, then why), individual characteristics (having had about the vaccine and cervical cancer, HPV knowledge), socio-economic factors(home area, number of members in the family, family head, access to media, being taught about HPV and cervical cancer at school, receiving vaccine from school, absence from school), Health system factors(estimated distance to facility, health worker recommendation, having seen posters at the facility about the HPV vaccine and cervical cancer, having visited the facility to get HPV vaccine, availability of the vaccine, availability of the health workers at the facility, exposure to outreach clinics). The outcome variable for this study was the self-reported receipt of the vaccine regardless of the number of doses.

### Sampling and data collection procedure

The study used a multistage sampling method whereby three sub counties or wards were first randomly selected out of five sub counties or wards in the city namely Ojwina, Lira Central, and Lira ward). There were over 20 primary schools in the three selected wards of which there were 8 public primary schools in Lira ward, 5 public primary schools in Adyel ward, and 3 public primary schools in Ojwina ward. For the second stage sampling, the two primary schools were selected out of the available schools in each of the three sub counties or wards. Six schools (namely; Barapwo primary school, Saving Grace primary school, Ober primary school, Lira Integrated primary school, Adyel primary school, and Lira Modern primary school) were randomly selected from the over 20 primary schools within the three sub counties from where the study participants were accessed. The total number of eligible pupils was established from which the participants were selected by probability sampling method.

Administration of the questionnaire with selected participants lasted between 25 and 40 minutes.

### Data management and analysis

While in the field every questionnaire was checked for completeness to ensure no missing data at the end of each interview process. A data entry screen with checks was created in SPSS version 23.0 to ensure no missing and out of range values are entered within the data set. Data were analysed using SPSS version 23.0. Continuous variables were summarised and presented as frequencies and percentages in appropriate tables during univariate analysis. Categorical variables were analysed using, cross-tabulation, and logistic regression at a 95% level of confidence during bivariate and multivariate analysis. Variables that were significant at bivariate analysis were tested for collinearity and included in the multivariate logistic regression model. The model fitness was checked using the Hosmer-Lemeshow test at p < 0.05 and the backward conditional logistic regression method was used to determine variables that were independently associated with the outcome variable. Variables that had p-values less than 0.05 were deemed significant at both bivariate and multivariate analysis.

## Results

### Demographic characteristics of school girls aged 9–14 years in Lira City, northern Uganda

A total of 245 girls aged 9–14 years were recruited in this study and all girls who were approached accepted to partake in this study giving us a response rate of 100%. The mean age of the girls was 12.11 (± 1.651) years. Majority of the participants; 219(89.6%) belonged to the Langi tribe, and 214(87.3%) stayed with their parents at home. In addition, majority of the participants, 181(73.9%) belonged to nuclear families and more than a third 97(39.6%) of school girls were Catholics. (Table 1).

### Uptake of HPV vaccine among school girls aged 9–14 years in Lira City, northern Uganda

In our study, 48 out of the 245 school girls were ever vaccinated against HPV 19.6% (95% CI 14.8–25.1). Among the vaccinated population, 3.7% reported to have received one dose, 9.8% had received two doses and 5.3% had received three doses of the HPV vaccine. However, of those who had never received the HPV vaccine, 62(43.1%) reported having no idea about the HPV vaccine as the reason to why they were not vaccinated.

### Bi-variate analysis of the Individual factors associated with uptake of HPV vaccine among school girls aged 9–14 years in Lira City, northern Uganda

In binary logistic regression, the factors that were found statistically associated with uptake of HPV vaccine at p value < 0.05 were; age of participants 12–14 years (p = 0.001), ever heard about the HPV vaccine (p ≤ 0.001), ever heard about cervical cancer (p ≤ 0.001), being taught about cervical and HPV at school (p ≤ 0.001) and in-school vaccination (p ≤ 0.001), and those who ever sought HPV vaccine from hospital (p ≤ 0.001) (Table 1).

**Table 1:** Demographic characteristics and the individual factors associated with HPV vaccine uptake among school girls in Lira City, northern Uganda

Characteristics	Frequency n(%)	Ever received HPV vaccine n(%)		cOR(95%CI)	*P*-value
		yes	No		
**age**					0.001*
9–11 12–14	81(33.1) 164(66.9)	5(2.0) 43(17.6)	76(31.0) 121(49.4)	Ref 5.40(2.04–14.24)	
**religion**					0.067
catholic anglican others	97(39.6) 93(38.0) 55(22.4)	26(54.2) 15(31.3) 7(14.6)	71(36.0) 78(39.6) 48(24.4)	Ref 0.53(0.26–1.07) 0.39(0.16–0.99)	
**tribe**					0.282
langi others	219(89.4) 26(10.6)	45(93.8) 3(6.3)	174(88.3) 23(11.7)	Ref 0.50(0.15–1.75)	
**family type**					0.207
nuclear family extended family	181(73.9) 64(26.1)	32(66.7) 16(33.3)	149(75.6) 48(24.4)	Ref 1.55(0.78–3.07)	
**ever heard of HPV vaccine**					≤ 0.001*
no yes	172(70.2) 73(29.8)	1(0.4) 47(19.3)	170(69.7) 26(10.7)	Ref 307.30(40.63–2324)	
**ever heard about cervical cancer**					≤ 0.001*
no yes	176(71.8) 69(28.2)	27(11.0) 21(8.6)	11(4.5) 186(75.9)	Ref 108.10(31.16–375.22)	
**health seeking behavior**					0.600
Traditional/herbal biomedical	42(17.1) 203(82.9)	7(2.9) 41(16.7)	35(14.3) 162(66.1)	Ref 1.26(0.52–3.05)	
**taught about cervical cancer at school**					0.001*
no yes	193(78.8) 52(21.2)	12(4.9) 36(14.7)	181(73.9) 16(6.5)	Ref 33.94(14.80–77.79)	
**ever received any vaccine at school**					< 0.001*
no yes	87(35.5) 158(64.5)	4(1.6) 44(18.0)	82(33.6) 114(46.7)	Ref 7.91(2.74–22.89)	
**ever sought HPV vaccine at hospital**					< 0.001*
no yes	209(85.3) 36(14.7)	21(8.9) 27(11.4)	179(75.8) 27(11.4)	Ref 25.57(10.61–61.62)	
**staying with parents**					0.354
no yes	31(12.7) 214(87.3)	8(3.3) 40(16.3)	23(9.4) 174(71.0)	Ref 0.66(0.28–1.58)	

*P-value < 0.05 statistically significant, cOR-crude odd ratio

### Health system factors associated with uptake of HPV vaccine among school girls aged 9–14 years in Lira City, northern Uganda

In binary logistic regression, the factors that were found statistically associated with uptake of HPV vaccine at p-value < 0.05 were; distance to the health facility (p = 0.036), availability of HPV vaccine at the hospital (p = 0.002), health workers recommendation (p ≤ 0.001, and exposure to outreach clinics (p < 0.001) (Table 2).

**Table 2:** Bivariate analysis of Health System factors associated with HPV vaccine uptake among school girls aged 9–14 years in Lira City, northern Uganda

Characteristics	Ever received HPV vaccine n(%)		cOR(95%CI)	*P*-value
	yes	No		
**distance to the facility**				0.036*
near far not sure	36(14.7) 11(4.9) 1(0.4)	107(43.7) 75(30.6) 15(6.1)	Ref 0.44(0.21–0.91) 0.19(0.03–1.55)	
**recommendation from health worker**				< 0.001*
no yes	10(4.1) 38(15.5)	178(72.7) 19(7.8)	Ref 35.60(15.34–82.63)	
**cervical cancer poster awareness**				< 0.001*
no yes	26(10.6) 22(9.0)	166(67.8) 31(12.7)	Ref 4.53(2.28–8.99)	
**availability of vaccines**				0.002*
no yes	3(8.3) 23(63.9)	7(19.4) 3(8.3)	Ref 17.89(2.93–109.33)	
**exposure to outreach clinics**				≤ 0.001*
no yes	28(11.4) 20(8.2)	185(75.5) 12(4.9)	Ref 11.01(14.86–24.97)	

*P-value < 0.05 statistically significant, cOR-crude odds ratio

### Predictors to uptake of HPV vaccine among school girls aged 9–14 years in Lira City, northern Uganda

Eleven variables were significantly associated with uptake of HPV vaccination at bivariate analysis but five variables were included in the multivariate logistic regression model. This is because we analysed for multi-collinearity between the independent variables and found six of the variables (perceived distance from the health facility, perceived availability of vaccines, ever sought HPV vaccines at school, ever heard of HPV vaccines, ever heard about cervical cancer, and ever received any vaccine at school) exhibited multi-collinearity, thus eliminated from the multivariate regression model. Hence, the predictors that were found to be independently associated with the uptake of HPV vaccine among school girls include; recommendation from the health worker [aOR 9.09, 95% CI (3.19–25.88), P ≤ 0.001], ever taught about cervical cancer at school [aOR 12.56, 95% CI (4.60–34.28), p ≤ 0.001], and exposure to community outreach clinics (aOR 4.41, 95% CI (1.37–14.19), p = 0.013] as shown in (Table 3) below.

**Table 3:** Multi-variate analysis of predictors to uptake of HPV vaccine among school girls aged 9–14 years in Lira City, northern Uganda

Characteristics	Ever received HPV vaccine n(%)		cOR(95%CI)	aOR(95%CI)	*P*-value
	Yes	no			
**Age**					0.350
9–11 12–14	5(2.0) 43(17.6)	76(31.0) 121(49.4)	Ref 5.40(2.04–14.24)	Ref 1.98(0.47–8.48)	
**recommendation from health worker**					≤ 0.001*
no yes	10(4.1) 38(15.5)	178(72.7) 19(7.8)	Ref 35.60(15.34–82.63)	Ref 9.09(3.19–25.88)	
**cervical cancer poster awareness**					0.842
no yes	26(10.6) 22(9.0)	166(67.8) 31(12.7)	Ref 4.53(2.28–8.99)	Ref 0.89(0.29–2.73)	
**taught about cervical cancer at school**					≤ 0.001*
no yes	12(4.9) 36(14.7)	181(73.9) 16(6.5)	Ref 33.94(14.80–77.79)	Ref 12.56(4.60–34.28)	
**exposure to outreach clinics**					0.013*
no yes	28(11.4) 20(8.2)	185(75.5) 12(4.9)	Ref 11.01(14.86–24.97)	Ref 4.41(1.37–14.19)	

*P-value < 0.05 statistically significant, cOR-crude odds ratio, aOR-adjusted odds ratio. Note: Six of the significantly associated independent variables from the bivariate analysis (namely (perceived distance from the health facility, perceived availability of vaccines, ever sought HPV vaccines at school, ever heard of HPV vaccines, ever heard about cervical cancer, and ever received any vaccine at school) were dropped from the multivariate regression model because they expressed multi-collinearity with other independent variables. Thus, these variables cannot independently predict the dependent variable

## Discussion

Our study found that just about one in five (19.6%) of the school girls aged 9–14 years in Lira city northern Uganda had received HPV vaccine. Our finding conforms with the result of a previous similar study conducted in Lira District, northern Uganda where uptake of the HPV vaccine was found to be 17.61% [17]. Similarly, our finding is close with the national HPV vaccine coverage among adolescent girls where uptake of the HPV vaccine was estimated at 22% [18]. The low HPV vaccine uptake reported in this study could be attributed to the negative attitude towards the vaccine [19], limited school and vaccination program resources, competing priorities within the school setting, and poor knowledge about HPV among teachers [20–22]. This finding implies that the current Ministry of Health Ugandan policy on school-based HPV vaccination program that targets school girls in primary school grade five regardless of their age [25] is not reaching many of the girls aged 9–14 years with the HPV vaccine as per the WHO recommended primary target [8]. According to the World Health Organization (WHO), the primary target age group for HPV vaccination is 9–14 years old, with a focus on vaccinating girls before they become sexually active [23]. Girls below the age of 9 are not recommended to receive the HPV vaccine due to insufficient data on safety and efficacy in this age group [24]. Similarly, girls above the age of 14 are not recommended to receive the HPV vaccine due to diminishing benefits and potential for reduced efficacy. This is because the vaccine is most effective when given before the onset of sexual activity and exposure to HPV. In addition, older girls may have a higher chance of already being infected with HPV, thus reducing the effectiveness of the vaccine.

As a cost-effective and easy to implement strategy to ensure girls aged 9–14 year old are reached with the HPV vaccine, the current policy of the Ugandan Ministry of Health recommends that, HPV vaccination be administered to school girls in primary school grade five regardless of their age [25]. However, this policy may not be effective in reaching all the eligible girls aged 9–14 years, as some may be older than 14 years or have dropped out of school as further confirmed by our study in a northern Ugandan setting where school-based delivery is the mainstay mechanism of HPV vaccination that found only 19.6% of these girls recommended age 9–14 years were reached with the HPV vaccine. Therefore, there is need for widening of the primary school grade of the girls to be targeted for the school-based HPV vaccination program from just primary school grade five only to grades four to six plus the 9–14 years age confirmations to reach the WHO recommended the primary target and secondary target for HPV vaccine [22, 23].

Furthermore, our study found that school girls who were taught about cervical cancer at school had thirteen odds of receiving HPV vaccination compared to their counterparts. This association could be due to the increased awareness and knowledge on cervical cancer, HPV vaccine to prevent cervical cancer and related health impact of cervical cancer on one’s life following the learning about cervical cancer in school [26, 27]. These findings concur with the results of a similar previous study conducted in Mbale District; eastern Uganda and Ethiopia where adolescent girls who received adequate information on the HPV vaccine had six and three odds respectively of utilizing the HPV vaccine compared to their counterparts [28, 29].This could be explained in a way that those who had heard about the vaccine and had knowledge about the HPV vaccine could have acknowledged its benefits which provoked them to go for the vaccine. The implication of the finding is that the ministry of health and related stakeholders in Uganda, sub–Saharan Africa and other developing countries should prioritize programs that create awareness about the HPV vaccine to optimize its uptake among young girls aged 9–14 years.

Our study also found that school girls aged 9–14 years in Lira City northern Uganda, who received a recommendation for HPV vaccination from health workers had nine odds of receiving the HPV vaccine compared to their counterparts. These findings conform to results from a previous study carried out in Mulago Hospital, Central Uganda where health worker recommendation was one of the factors associated with the timely completion of the HPV vaccination among girls aged 9–14 years [30]. This association could be due to the societal trust held to the information provided by the health care worker and the efforts of health workers towards raising awareness of HPV vaccine among adolescent girls [27, 29, 31]. This highlights the importance of health workers’ recommendations and or referrals in improving the uptake of the HPV vaccine, whereby with more recommendations from them, more girls would get vaccinated. The findings imply that the health education materials that health workers in Uganda, sub–Saharan Africa and other developing countries use to provide health education in facility-based and or community-based settings should be revised to include HPV vaccine messages, recommendations and or referrals [29].

Our study also found that school girls who were exposed to outreach clinics were having four odds of receiving HPV vaccine compared to their counterparts. This association could be due to synergies built between HPV vaccination and the existing health programs such as other immunization services, deworming, and HIV testing services, which are widely accepted by the community and the in schools [1, 32, 33]. The finding imply that community outreaches for immunization and other health promotional services which are part and parcel of primary health care activities of health facilities in Uganda, sub Saharan Africa and other developing countries should always include HPV vaccines to increase the HPV vaccination uptake among young girls.

## Study limitations and strengths

This study used a small sample size of 245 school-going adolescent girls. This could have affected statistical power thus impacting on the reliability of this study. However, the results are comparable with one of the previous cross-sectional study conducted in Lira District with a sample size of 460 female adolescents which also found a low (17.61%) uptake of HPV vaccine among female adolescents [17]. The study only employed a quantitative approach and hence missed out on the qualitative data which always complements the quantitative data. However, the results of this study can be used as an inference for future research studies. Self-reported bias of the HPV vaccination uptake since we didn’t confirm vaccination by vaccination card.

## Conclusion

The study found that one in five of the school girls in Lira City, northern Uganda received HPV vaccine. Girls who were taught about cervical cancer at school, exposure to outreach clinics and received health worker recommendation were more likely to receive HPV vaccine than their counter parts. The Ministry of Health should strengthen school based cervical cancer education, awareness raising about HPV vaccination and health worker recommendations to improve HPV vaccine uptake among school girls in Uganda.

There is also need to empower the teachers with the information about HPV and cervical cancer such that they can easily pass it on to girls in school and also be champions in their communities to ease dissemination of information.

## Data Availability

The data sets used and/or analyzed during the study are available from the corresponding author on reasonable request.
